# The complexities of global health negotiations: Power dynamics and the politics of the pandemic agreement

**DOI:** 10.1371/journal.pgph.0003841

**Published:** 2024-11-07

**Authors:** Chiamaka P. Ojiako, Tamrat Shaweno, Nebiyu Dereje, Jackline Kiarie, Benjamin Djoudalbaye, Morẹnikẹ Oluwatoyin Folayan, Maha El Rabbat, Amr Ahmed Ramadan, Jean Kaseya, Nicaise Ndembi

**Affiliations:** 1 The Robert F. Wagner Graduate School of Public Service, New York University, New York, New York, United States of America; 2 Africa Centres for Disease Control and Prevention (Africa CDC), Addis Ababa, Ethiopia; 3 Amref Health Africa, Nairobi, Kenya; 4 Department of Child Dental Health, Obafemi Awolowo University, Ile-Ife, Nigeria; 5 Faculty of Medicine, Cairo University, Giza, Egypt; 6 Vice-Chair of the Intergovernmental Negotiating Body to Draft and Negotiate the new Pandemic Agreement, Cairo, Egypt; London School of Economics (LSE), UNITED KINGDOM OF GREAT BRITAIN AND NORTHERN IRELAND

## Introduction

Collaborative efforts are necessary to address threats to global health security and economies [1]. Such collaborative efforts may require establishing formal agreements to regulate stakeholders’ actions and inactions [2]. Formal binding agreements is usually challenging to reach because of the complexity of negotiations and debates, steeped in power dynamics, diverse actor interests, and alliances that influences the prioritization of fairness, justice, and equity, ensuring equitable access to medicines and vaccines [3].

The COVID-19 pandemic highlighted gaps in global health governance and the need for collaboration. In December 2021, WHO Member States established an intergovernmental negotiating body (INB) to draft an agreement on pandemic prevention, preparedness, and response to ensure better preparedness and coordinated responses for future pandemics [4]. The negotiation process has, however, faced challenges due to the complexity of involving diverse countries with varying priorities, resources, differing healthcare infrastructure development and conflicting interests. Yet, the pandemic agreement must address issues of sovereignty and national autonomy, as countries may hesitate to commit to binding obligations that limit their independent public health decisions. Balancing global coordination with national sovereignty is challenging.

Despite these obstacles, the INB’s establishment and ongoing negotiations are crucial steps toward improving global pandemic preparedness, enhancing global health security and preventing future pandemics remain urgent priorities. This piece aims to spark discourse on international politics and political economy of global health factors influencing global health negotiations.

### Influence of political power and dynamics in global health decisions

Global health cooperation is rarely the sole motivator behind debates and negotiations for agreements on global health emergencies. The primary funders of global health initiatives, often exert considerable influence over international health policies and priorities. As demonstrated during COVID-19, there were political conflicts affecting vaccine rollout with countries with similar interests forming alliances and networks to amplify their voices and protect their interests [5] leading to vaccine nationalism [6]. There was also the intertwine of the monopolization of vaccine and medicine production by industries that prioritize profit and the monopolization of access by high-income countries’ purchasing power. The delayed Ebola response in West Africa, and the slowness of the mpox research and response in Africa [7] are other examples.

In an attempt to build on lessons learnt during the pandemic, the World Health Assembly requires input from multiple actors and the 194 Member States in the INB proceedings. However, this intention of the World Health Assembly Member States conflicts with the interests of powerful pharmaceutical companies that need to relinquish control over medicine and vaccine access motivated, and their home countries that benefit from the current intellectual property (IP) protections and market-driven health solutions. This stalled progress on addressing contentious areas of the agreement like Pathogen Access and Benefit Sharing, IP, and financing [8].

After multiple iterations, the INB reached a consensus in various articles, including articles that addressed equitable access to medical countermeasures, the creation of a global health emergency preparedness and response fund, transparent sharing of outbreak information, support for low and middle-income countries, and a network of supply chains [9]. The latest draft of the WHO pandemic treaty still includes a language for IP rights waiver that is still a cause of concern for many [10].

### The pandemic agreement–Lessons on navigating global health politics and the critical role of diplomacy

The Pandemic Agreement negotiation highlights the importance of global health politics and the role of money in decision-making evident in the dynamics of local COVID-19 vaccine manufacturing. During the pandemic, high-income countries and large pharmaceutical companies were skeptical of local manufacturing in low- and middle-income countries, citing concerns about technical capacity, quality standards, intellectual property rights, and market competition. However, as vaccine supply shortages were addressed and the benefits of local production became clear, the narrative shifted [11]. Pharmaceutical companies sought to expand markets by partnering with local manufacturers, aligning with strategies to increase global reach and profitability. Governments and international organizations, facing pressure to address vaccine distribution inequities, supported local manufacturing to promote global health equity and counter vaccine nationalism [12]. Successful local manufacturing and technology transfers built confidence in these facilities, reducing skepticism. Increased international support for local manufacturing, seen in initiatives like the COVAX Facility and collaborations between WHO, Gavi, and CEPI, was part of the observed shift. This evolution highlights how power dynamics, market forces, and ethical considerations intersect in global health cooperation to influence decision-making and shaping the landscape of international health cooperation.

Recognizing when politics, diplomacy, or both are at play in ways that can influence collaboration and negotiations for better pandemic preparedness is crucial [13]. Acknowledging the influence of high-income countries and large pharmaceutical companies in shaping pandemic preparedness, funding, and policy allows negotiators to ensure that the voices of low- and middle-income countries are heard. Understanding political and diplomatic stakes allows for transparency and crafting compromises when negotiating mutually beneficial agreements, fostering cooperation, resource sharing, and technology transfer. Its also important to understand that the concepts of justice and equity can be subjective and influenced by power and money and become obstacles in global health negotiations.

Yet, negotiating the Pandemic Agreement is crucial for securing funding and technical support from high-income countries and international organizations to allocate resources, facilitate technology transfer, and share knowledge, especially with low- and middle-income countries. It is essential to address these dynamics to ensure fair access to resources and effective implementation of policies and frameworks with adequate support from all stakeholders.

### What next for equity during pandemics

Pandemics highlighted the interconnectedness of our world and the need for a unified health crisis response. Achieving a global Pandemic Agreement is challenging, may take time but it is essential for strengthening international cooperation crucial for global health security, treating the costs as investments in future global health. All countries can be heard during negotiations. Equity-focused negotiations can lead to stronger health systems worldwide, especially in low- and middle-income countries, creating a resilient global health network.

Negotiations would require that the control over vaccine and medicine access move beyond the profit-driven models by the pharmaceutical industry often linked to the purchasing power of high-income countries. This can ensure vital health resources does not remain concentrated in the hands of powerful stakeholders. Equity in access to medicines and vaccines would also require a shift in how global health priorities are defined and acted upon. While protecting the financial viability of pharmaceutical companies is important, the public health impact of excluding low-income countries from timely access to vaccines must not be overlooked. Future pandemic agreements should emphasize the global public good over profit maximization, ensuring that all countries, regardless of income, have equal access to life-saving resources. It means redefining success not just by profit margins, but by how effectively global health systems can respond to the needs of the most vulnerable populations.

Addressing health by developing a cooperative framework for addressing complex global challenges disparities no nation can tackle alone, ensures all countries have the tools to combat pandemics, enhancing global stability and security. While ensuring equity in pandemic preparedness has significant upfront costs, the long-term economic benefits outweigh these expenses. Investing in equitable health measures is both an economic and moral imperative, reflecting our shared humanity and commitment to a more equitable world.
